# State-of-the-Art Mediastinal Staging in Non-Small-Cell Lung Cancer: Integration of Combined Endosonographic Techniques with Updated IASLC TNM 9th Classification

**DOI:** 10.3390/cancers18101666

**Published:** 2026-05-21

**Authors:** Omar Alkathiri, Moishe Liberman

**Affiliations:** Thoracic Surgery Department, Centre Hopitalier de l’Université de Montreal, Pavillon R, 900 Rue Saint Denis, Montreal, QC H2X 0A9, Canada

**Keywords:** non-small cell lung cancer, mediastinal staging, Endobronchial Ultrasound (EBUS), Endoscopic Ultrasound (EUS), EBUS-TBNA, EUS-FNA, combined Endosonography, IASLC TNM classification, lymph node staging, molecular profiling

## Abstract

The objective of this review is to evaluate the role of combined endobronchial and esophageal endosonography in mediastinal staging and to define its clinical implications within the proposed IASLC TNM 9th edition framework. Mediastinal staging remains a critical step in the management of non-small cell lung cancer (NSCLC), as it directly impacts treatment planning, surgical decision-making, and overall prognosis. For many years, mediastinoscopy was considered the standard approach; however, in routine practice, it has largely been replaced by less invasive techniques. Endobronchial Ultrasound (EBUS) and Endoscopic Ultrasound (EUS) have become widely adopted because they allow real-time sampling of lymph nodes with good accuracy and a low complication rate. In clinical settings, these techniques are often used together rather than separately, as each provides access to different nodal stations. This combined approach improves diagnostic yield and reduces the number of patients who require surgical staging. At the same time, recent updates in the IASLC TNM classification, including the proposed 9th edition, have introduced more detailed nodal categories, making accurate tissue confirmation even more important in daily practice. In this review, we summarize the current use of combined EBUS and EUS in mediastinal staging, focusing on their practical advantages, limitations, and roles across different clinical scenarios. We also discuss their relevance in the context of molecular testing and evolving treatment strategies. Despite their strengths, there are situations in which negative results should be interpreted with caution and confirmed surgically. Overall, these techniques have reshaped the approach to mediastinal staging and are now central to modern lung cancer care.

## Simple Summary

Mediastinal staging plays a central role in the management of non-small cell lung cancer (NSCLC), as it guides treatment decisions, determines surgical eligibility, and influences prognosis. Although mediastinoscopy has long been considered the reference standard, minimally invasive endosonographic techniques—namely endobronchial ultrasound (EBUS) and endoscopic ultrasound (EUS)—have become the preferred initial approach in many centers. When used together, these techniques allow more comprehensive access to mediastinal lymph nodes and improve diagnostic performance while maintaining a favorable safety profile. At the same time, recent updates in the IASLC TNM classification, particularly the proposed 9th edition, introduce more detailed nodal stratification, further underscoring the importance of accurate tissue-based staging. In this review, we discuss the evolving role of combined EBUS and EUS in mediastinal staging, highlight their clinical advantages compared with surgical methods, and explore their growing relevance in the era of molecular testing and personalized treatment in lung cancer.

## 1. Introduction

Mediastinal lymph node staging remains a cornerstone of lung cancer management, influencing treatment selection, surgical candidacy, and prognosis. Traditional surgical staging using mediastinoscopy has historically been considered the gold standard. However, minimally invasive endosonographic techniques have increasingly replaced surgical staging due to their superior diagnostic yield and improved safety profile. Furthermore, combining endobronchial ultrasound-guided transbronchial needle aspiration (EBUS-TBNA) with endoscopic ultrasound-guided fine needle aspiration (EUS-FNA) has demonstrated higher diagnostic yield than mediastinoscopy in certain settings, as it allows sampling of lymph nodes and metastases that are not accessible by mediastinoscopy. The European Respiratory Society (ERS) recommends the combined use of EBUS and EUS, rather than either modality alone, for mediastinal staging in lung cancer. The American College of Chest Physicians (ACCP) also recommends EBUS-TBNA, EUS-FNA or a combination of both over surgical staging as the initial diagnostic approach for mediastinal staging in non-small cell lung cancer (NSCLC) [1]. Precise mediastinal staging is essential for optimal management of NSCLC.

Concurrently, ongoing refinements of the IASLC TNM staging system have improved anatomic classification and prognostic accuracy. The 8th edition and the proposed 9th edition introduce more granular tumor size subdivisions, nodal stratification, and expanded metastatic categories, necessitating the integration of precise tissue-based staging techniques into clinical workflows.

## 2. Indications for Invasive Mediastinal Staging

Current staging recommendations support invasive mediastinal evaluation in patients with:

- Tumors larger than 3 cm;
- Centrally located tumors;
- PET-positive mediastinal lymph nodes;
- Enlarged mediastinal lymph nodes on CT imaging;
- Suspected N1 disease.

Patients with small, peripheral stage IA tumors and negative imaging findings may be considered for direct surgical resection without invasive mediastinal staging [2].

The ninth edition of the TNM classification for lung cancer introduces adjustments to nodal descriptors, specifically subdividing N2 disease into single-station (N2a) and multi-station (N2b) nodal metastases (see Table 1 and Table 2). Single-station N2 disease is associated with a more favorable prognosis than multi-station involvement [3]. This revision has significant implications for clinical practice and underscores the need for comprehensive mediastinal staging within a multidisciplinary framework.

## 3. Evolution of Mediastinal Staging

Imaging-based modalities, including computed tomography (CT) and positron emission tomography–computed tomography (PET-CT), provide essential structural and metabolic information but cannot establish a definitive histologic diagnosis. Both modalities demonstrate limited specificity and sensitivity for mediastinal lymph node assessment, particularly in differentiating malignant from inflammatory lymphadenopathy. Consequently, tissue confirmation remains necessary in many clinical scenarios [2,3,4,5,6,7,8,9,10]. The emergence of endosonographic staging, combining endobronchial and esophageal ultrasound (EBUS and EUS), has led to its adoption as the preferred initial minimally invasive staging modality in many centers. These techniques provide real-time ultrasound guidance for nodal sampling and allow access to both mediastinal and selected extrathoracic targets, including the adrenal glands, celiac lymph nodes, and the left hepatic lobe. Reported diagnostic performance includes:

- EBUS-TBNA sensitivity: up to 93% [11,12,13,14,15,16,17];
- EUS-FNA sensitivity: up to 89% [18,19,20,21,22,23,24];
- Combined EBUS/EUS sensitivity: approximately 92% [25];
- Negative predictive value: >90% [26].

These outcomes are comparable to those of surgical mediastinoscopy while offering a broader anatomic reach and reduced procedural morbidity [27,28,29,30].

## 4. Mediastinal Nodal Mapping and Endosonographic Access

**Figure 1:**
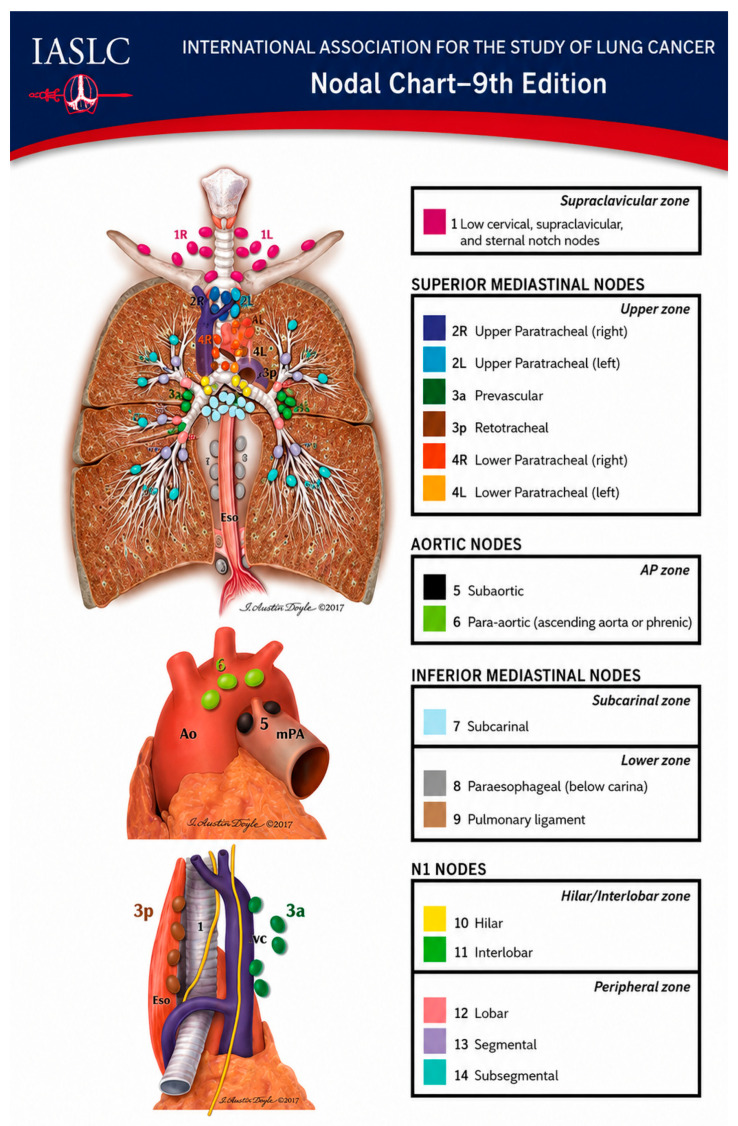
IASLC lymph node map (9th edition) illustrating the anatomical distribution of thoracic lymph node stations. The map includes supraclavicular nodes (station 1), superior mediastinal nodes (stations 2R/L, 3a, 3p, 4R/L), aortic nodes (stations 5 and 6), inferior mediastinal nodes (stations 7, 8, and 9), and N1 nodes including hilar and intrapulmonary stations (stations 10–14). This classification provides a standardized framework for the assessment of mediastinal and hilar lymph nodes in lung cancer staging. (copyrights agreement from IASLC).

Accurate identification of lymph node stations is essential for standardized and reliable staging. The IASLC nodal map (Figure 1) defines mediastinal, hilar, and intrapulmonary lymph node stations and provides a framework for systematic sampling. Evaluating all accessible lymph nodes using both EBUS and EUS can improve diagnostic and staging accuracy [31].

### 4.1. Stations Accessible by EBUS

The EBUS scope is a flexible echo bronchoscope that provides a field of view of 50° to 70° and generates images parallel to the shaft. According to ACCP guidelines, EBUS-TBNA is recommended as the preferred initial diagnostic modality for suspected N2 or N3 disease. If results are negative, surgical sampling should be considered for clinically suspicious nodes, and the selection of the initial diagnostic modality should depend on the operator’s expertise [1].

EBUS-TBNA allows reliable sampling of upper and lower paratracheal nodes (stations 2R/L and 4R/L), subcarinal nodes (station 7), hilar and interlobar nodes (stations 10, 11, and 12).

EUS can also be performed using the EBUS scope (EUS-B-FNA), and it improves sensitivity when added to EBUS [32]. EUS can access stations 2L, 4L, 5, 6, 7, 3P, 8R/L, 9R/L, Celiac axis lymph nodes and the left and right adrenal glands. Stations 2R and 2L may also be accessible if the nodes are enlarged. Hwangbo and colleagues reported that lymph nodes detected by EUS-B but missed by EBUS were not located in the inferior mediastinum; rather, they were primarily at stations 4L and 5. In this study, EUS was performed using an EBUS bronchoscope (EUS-B approach). In a prospective trial including 150 patients with NSCLC who underwent combined EBUS and EUS-B during the same procedure using a single bronchoscope (instead of a conventional EUS endoscope), the addition of EUS-B increased overall mediastinal staging sensitivity from 82% to 92%.

EUS-B provides access to nodal stations that are difficult or inaccessible with EBUS alone, particularly stations 5, 8, and 9, as well as the left adrenal gland, thereby enhancing the completeness and accuracy of mediastinal staging [33].

### 4.2. Stations Accessible by EUS

The linear EUS scope provides a wider field of view than the EBUS scope, offering a 180° view. EUS-FNA complements EBUS by enabling access to inferior mediastinal nodes (stations 8 and 9), aortopulmonary window nodes (stations 5 and 6), adrenal glands, celiac axis lymph nodes, and the left hepatic lobe. EUS also facilitates access to smaller station 2L and 4L lymph nodes compared to EBUS. Transesophageal lung biopsy with EUS can be performed during the same procedure and appears to be safe for patients with central tumors near the esophagus [34]. In a study of 138 patients who underwent both EBUS and EUS, EUS demonstrated higher sensitivity for detecting malignancy at lymph node stations 5, 6, and 7 [35]. For patients with adrenal involvement, a dedicated EUS scope enables evaluation of both mediastinal nodes and adrenal glands in a single procedure. This approach reduces patient discomfort, risk, and cost [36].

EUS serves as a valuable complementary approach for perimediastinal lung lesions when conventional biopsy methods are infeasible or associated with excessive risk, and it expands the diagnostic capabilities available for individualized lung cancer management. In a retrospective cohort analysis of 55 patients carefully selected with intraparenchymal pulmonary nodules suspicious for malignancy, in whom standard biopsy techniques were either unsuccessful or deemed inappropriate. All procedures were performed using real-time endoscopic ultrasonography with a 22-gauge needle, ensuring precise lesion localization and controlled tissue acquisition. Adequate cytologic or histologic sampling was achieved in 94.5% of cases, with a corresponding diagnostic accuracy and sensitivity of 94.5%, confirming the reliability of this technique, with no procedure-related morbidity, including pneumothorax, pleural effusion, or esophageal complications [34]. Nasir et al. emphasize that EUS-guided lung biopsy is not intended to replace established diagnostic modalities but rather to serve as a complementary tool within a multidisciplinary diagnostic algorithm. Its greatest clinical value lies in high-risk patients, in cases where histologic confirmation is required prior to oncologic therapy.

## 5. Advantages of Combined Endosonographic Staging

Combining EBUS and EUS improves mediastinal coverage and diagnostic sensitivity compared with either modality alone. Meta-analytic data indicate that sensitivity gains of up to 21% may be achieved when both techniques are employed systematically [31,37]. This comprehensive approach reduces false-negative results and may limit the need for confirmatory surgical staging. Comparative studies have shown that combining endobronchial and esophageal ultrasound techniques improves staging performance compared with either modality alone. Reported data indicate an approximate 11% improvement in sensitivity when EBUS-TBNA is integrated with EUS-based sampling for lung cancer staging [38]. In a prospective cohort of 166 patients, outcomes from combined endosonographic staging were compared with mediastinoscopy, with final surgical pathology serving as the reference standard. The combined approach identified advanced nodal or metastatic disease (N2, N3, or M1) in 14% of cases initially classified as negative by mediastinoscopy, thereby preventing unnecessary surgical procedures [39]. The diagnostic performance of the combined technique was high, with a sensitivity of 91%, specificity of 100%, a negative predictive value of 96%, and an overall accuracy of 97%.

Conventional mediastinoscopy mainly accesses paratracheal and subcarinal lymph nodes. However, several important regions—including the paraesophageal, aortopulmonary window, para-aortic, and inferior pulmonary ligament stations—are not routinely accessible with this method [40,41,42,43]. Moreover, the inferior portion of the subcarinal space may remain inaccessible. Nodal metastases that go undetected during mediastinoscopy are most often located in these less accessible areas [44,45].

Complications are uncommon with combined EBUS/EUS mediastinal staging. The overall complication rate is approximately 1–2%, with a reported mortality of 0.04%. Most complications are minor (e.g., transient hypoxia and minor bleeding), while severe adverse events are rare.

In comparison, mediastinoscopy carries a higher morbidity, with an overall complication rate of approximately 2–3%. Reported mortality is approximately 0.08%, most commonly related to major vascular injury or catastrophic bleeding. Therefore, combined endosonographic staging (EBUS/EUS) is associated with lower morbidity and approximately half the mortality rate compared with mediastinoscopy, while maintaining high diagnostic accuracy [46,47].

### 5.1. Practical Implications of IASLC TNM 9th Edition for Endosonographic Staging

Recent revisions to the TNM Classification System for Endosonography, as developed by the IASLC (International Association for the Study of Lung Cancer) have new definitions and implications for staging through the use of endosonography. One of the primary changes in this new classification system is subdividing N2 (mediastinal) involvement into either N2a (involvement of a single nodal station) and N2b (involvement of multiple nodal stations). This added specificity will help create a more precise assessment of how involved the disease is and help direct clinical decisions.

Current evidence-based guidelines recommend minimally invasive mediastinal staging using endosonographic techniques, including endobronchial ultrasound-guided transbronchial needle aspiration (EBUS-TBNA) and/or endoscopic ultrasound-guided fine-needle aspiration (EUS-FNA), as the preferred initial approach for mediastinal nodal evaluation in patients with suspected or confirmed non-small cell lung cancer. Surgical mediastinal staging is generally reserved for cases with negative endosonographic findings despite persistent clinical or radiologic suspicion of nodal disease [3,28]. In summary, knowing whether the NSCLC is classified as N2a or N2b will affect how one proceeds with systematic sampling of all accessible lymph node stations within the mediastinum for purposes of determination between either being N2a vs. N2b. Systematic sampling must include bilateral mediastinal nodal station assessment (e.g., R4/L4, 7) regardless of the imaging studies concluding that the patient’s disease is limited in extent. It is not sufficient to just obtain targeted samples from only the radiological suspicious nodal stations; therefore, obtaining subliminally contaminated (non-targeted) samples will result in false-negative nodes and/or a patient being falsely staged as N2a instead of N2b.

With regard to how procedures are performed, regardless of whether a given lymph node station is classified as N2a or N2b, the technique of performing multiple needle passes will be performed in a similar manner. Current evidence suggests three being optimal for producing a diagnostic yield for a node; however, it is recommended that there should be an increase in the total number of sampled stations in order to ensure all stages and correct classification of nodes have been sampled.

Ensuring appropriate sampling is also important since patients with N2b disease will generally have a poorer prognosis compared to patients with an N2a disease and this may affect the patient’s eligibility for surgical treatment. However, some selected patients with N2a may be eligible for resection even when multimodally treated. Most patients with N2b will not be surgical candidates and will therefore have their treatment directed towards non-surgical modalities.

The negative results from an endosonographic staging should be considered in the context of this updated paradigm. When it comes to negative endosonographic results, be sure to examine those results closely as differences in results between N2a and N2b may lead to different treatments. Additionally, if a patient has ongoing clinical or imaging concerning for mediastinal involvement, it is necessary to do an additional surgical staging with mediastinoscopy or thoracoscopy. This is especially important for candidates that want curative surgical Intent as being understaged can lead to improper treatment.

There are also implications of TNM 9th edition applicability beyond just surgery in the treatment planning process. For example, patients considered for stereotactic body radiation therapy (SBRT) need to be accurately determined to not have nodal disease. Undetermined inclusion would cause severe under staging of patients leading to the wrong treatment decisions. Therefore, complete endosonographic staging will be critical in the determination of appropriate patients for SBRT.

Overall, the 9th edition TNM classification underlines the importance of systematic, rather than selective, mediastinal staging using collective endosonographic techniques.

### 5.2. Clinical Algorithm for Endosonographic Mediastinal Staging

The way that a person goes about getting tested for any type of cancer is important to help him or her receive the correct diagnosis. When patients have suspected or confirmed NSCLC, patients have structure to follow to help improve the diagnosis and help in getting the right treatment.

The initial way to determine if there are any cancers in the chest (mediastinum) is with a cross sectional imaging from a CT scan and or from a PET scan. If there is a suspicion of there being lymph nodes in the chest by the CT scan or PET scan, the first way to further evaluate the lymph nodes in the chest would be by either an EBUS or EUS.

If the endosonographic sampling has been confirmed to be a positive lymph node metastasis, then the extent of the nodal involvement would be used to stage the disease based on this information and help to determine how to manage the disease. Differentiating whether there are single-station N2a or multi-station N2b are factors in determining if a patient will be a surgical candidate and treatment pathway.

If the endosonographic results are negative but there is still high clinical or radiological suspicion, mediastinoscopy to surgically stage the patient should be carried out to either confirm or exclude false negative results.

Where it has been determined that the patient has no mediastinal involvement and the staging has been adequate, treatment will depend on the patient’s tumor characteristics and overall clinical condition. If the patient will have a ‘cure’, then surgical resection can be performed; if the patient does not have a ‘cure’, then stereotactic body radiation therapy (SBRT) can be used since the patient is not a surgical candidate.

In patients being considered for SBRT, it is essential that accurate nodal staging has been performed, as if there is undetected nodal disease, there is a likelihood of undertreatment. For these reasons, it is recommended that a complete endosonographic staging evaluation be done prior to the initiation of any type of definitive therapy.

## 6. Special Clinical Scenarios

### 6.1. Radiologically Normal Mediastinum

Occult nodal metastases may be present despite normal CT or PET findings. Combined endosonographic staging improves detection of subclinical nodal disease and may reduce the need for surgical staging when results are negative. Herth and colleagues evaluated 100 patients with normal lymph nodes on CT who underwent EBUS for preoperative staging. EBUS identified 19 of 20 occult metastases not detected by CT scans [48]. In a separate study by the same group, EBUS-TBNA detected 8 of 9 occult lymph node metastases in 97 patients with PET-negative mediastinum [49]. Shingyoji et al. reported findings from 113 patients who underwent preoperative EBUS staging. Of these, 20 patients (17.6%) had N2 disease, but EBUS-TBNA identified occult disease in only 7 of them [50]. Ong et al. analyzed 220 patients who received preoperative EBUS staging, identifying 49 patients (22%) with occult metastases; EBUS detected 18 (8%) of these 49 cases [51].

### 6.2. Restaging After Neoadjuvant Therapy

Post-treatment fibrosis and necrosis may reduce sampling sensitivity [52]. Negative endosonographic findings following induction therapy should be interpreted with caution and may require surgical confirmation based on the recommendations of European Society of Thoracic Surgeons (ESTS) if clinical management decisions depend on nodal clearance. In a recent study by Cetinkaya et al., 44 patients with 73 lymph nodes were biopsied after neoadjuvant therapy. EBUS-TBNA detected malignant cells in 23 patients (57.5%) and 25 lymph nodes (34.2%). No nodal metastasis was found in 21 patients (42.5%) and 48 lymph nodes (65.8%). All patients with negative EBUS-TBNA results underwent mediastinoscopy (*n* = 9) or surgery (*n* = 12). Metastatic involvement was subsequently identified in 5 of 21 patients (23.8%) and 6 of 48 lymph nodes (12.5%). The sensitivity, specificity, positive predictive value, negative predictive value, and overall accuracy of EBUS-TBNA were 82.1%, 100%, 100%, 76.2%, and 88.6%, respectively. Negative EBUS results following induction therapy should therefore be confirmed with invasive procedures such as mediastinoscopy and/or thoracoscopy [50]. The sensitivity of EUS-FNA for restaging after chemoradiation is approximately 44% with a false negative rate of 58% [53]. Re-staging should only be performed in cases where it changes surgical management such as in new nodal or contralateral (N3) nodal disease due to progression during induction.

### 6.3. Stereotactic Body Radiation Therapy (SBRT) Planning

EBUS-TBNA plays a critical role in excluding occult N1 disease prior to stereotactic body radiation therapy, leading to stage migration and treatment modification in a substantial proportion of patients. Reported studies show a stage shift of 16–19% in patients considered for SBRT [54,55].

### 6.4. Risks and Limitations of Endosonographic Staging

Endoscopic ultrasound and endobronchial ultrasound-guided transbronchial needle aspiration are generally non-invasive techniques to sample tissue under imaging guidance; however, there do exist some complications associated with these techniques. Clinicians should be aware of potential complications, as well as limitation(s) related to the diagnostic performance of these techniques.

Bleeding is an uncommon but potential complication, primarily at the site of aspiration on the needle. Major hemorrhagic events, such as required blood transfusions, are rare; however, may occur in patients with coagulopathy or when a highly vascular structure has unintentionally been aspirated (e.g., thoracic aorta) [46,47].

Although infectious complications are uncommon, they can occur (i.e., mediastinitis, abscess formation) and tend to occur more frequently when aspirating a cystic lesion or necrotic lymph node. Careful selection of patients and strict adherence to sterile technique are important for reducing the risk of infection in addition to other types of infections [56,57,58,59].

There is a risk for hypoxia to occur during these procedures, particularly if patients have impaired pulmonary function or are under prolonged sedation. Appropriate monitoring and use of sedating agents will minimize the risk of hypoxia to patients [46].

The false-negative rate of staging via endoscopic ultrasound (EUS) is a significant drawback, with sampling errors, the operator’s level of experience, necrotic lymph nodes (and post-treatment fibrosis) all working to reduce the diagnostic sensitivity [52]. Therefore, in patients where the pre-test probability suggests a high likelihood of having lymph node disease, negative results should be viewed with caution, and confirmation should be obtained through surgical staging (if possible).

Accessing various lymph node stations; especially 5 (the aortopulmonary window) and 6 (the para-aortic region), is still very difficult procedurally. There are many advanced techniques using either EUS or modified endobronchial ultrasound (EBUS) that have been proposed, but they add complications to the procedure and the associated risks (e.g., vascular injury due to their proximity to very major vessels such as the aorta) increase [60,61,62]. Therefore, any sampling of these stations should occur only in experienced centers with appropriate expertise.

## 7. The Role of Endosonography in the Context of Molecular Markers

The amount of tissue sampled is critical, and studies on EBUS have shown promising results [63,64,65,66,67,68,69]. The adequacy of EBUS-TBNA for molecular analysis depends on factors such as sample size, tumor necrosis, nodal micrometastasis, and specimen contamination [70]. Molecular analysis is feasible in most EBUS and EUS samples, with reported success rates between 89% and 98% [34,35,36,39,66,71,72,73]. Obtaining sufficient tissue is essential for PD-L1 testing, which is now standard in the management of NSCLC. Sakakibara and colleagues compared EBUS-TBNA with transbronchial biopsy (TBB) for PD-L1 expression and assessed concordance with surgical specimens. EBUS-TBNA, TBB, surgical specimens, and lymph node metastases generally showed similar PD-L1 positivity rates. EBUS-TBNA was more reliable than TBB in both the number and quality of tumor cells collected [73]. Further studies are needed to confirm the value of endosonography in this context.

Transbronchial cryobiopsy using EBUS (cryo-EBUS) is emerging as a complementary technique capable of retrieving larger, well-preserved histologic specimens. In a case series done by Genova et al., five consecutive patients with thoracic malignancies or suspected lymphoproliferative disease underwent cryobiopsy for subcarinal node (station 7) and showed better tissue architecture preservation and greater material volume compared to cytology, in 3 out of 5 cases cryo-EBUS provided complete lymphoma histology, larger tissue samples for molecular profiling in NSCLC and full molecular testing, including PD-L1.

This supports next-generation sequencing and biomarker testing (EGFR, ALK, ROS1, BRAF, MET, RET, NTRK, KRAS, PD-L1) using endosonographic techniques. Adequate sampling is critical for therapy selection, including immunotherapy and targeted treatments [74].

## 8. Technical Aspects of the Procedure

All procedures were performed under conscious sedation or general anesthesia following institutional protocol. An initial airway assessment was conducted using whitelight bronchoscopy to facilitate mucosal anesthesia and evaluate endobronchial anatomy before ultrasound-guided staging. EBUS was then performed, followed by EUS when additional nodal access was needed [75]. Consistent transducer–wall contact, minimal probe angulation, and continuous suction during needle aspiration helped ensure optimal sonographic image quality. A standardized, stepwise approach to mediastinal staging was implemented. Sampling began with lymph node stations on the side opposite the primary tumor (N3), followed by ipsilateral mediastinal stations (N2), and then ipsilateral hilar nodes (N1) [76]. The sequence was adjusted in certain cases based on tumor location and procedural findings to reduce the risk of cross-contamination.

### 8.1. Lymph Node Station Localization

Mediastinal lymph node stations were defined according to established anatomical landmarks and mapped using both EBUS-guided transbronchial needle aspiration (TBNA) and EUS-guided fine-needle aspiration (FNA) techniques [77].

Highest mediastinal nodes (Station 1) cover the area between the inferior border of the cricoid cartilage and the thoracic inlet, bounded in front by the manubrium and on the sides by the clavicles. Access was obtained bronchoscopically using the midline of the trachea as a reference; the left-sided part was also accessible via the esophagus, but the suggested borders were not visible on EUS.

Upper paratracheal nodes (Station 2) on the right side (2R) were approached along the lateral tracheal wall, guided by the innominate vein above its junction with the superior vena cava. Left-sided nodes (2L) were evaluated relative to the upper margin of the aortic arch, using the trachea as the central landmark.

Prevascular and retrotracheal nodes (Station 3) are subdivided into anterior (3A) and posterior (3P) compartments, extending from the thoracic inlet to the carina. The anterior part, located ventral to the great vessels, was visualized on EBUS but not sampled by EUS due to airway interference. The posterior part, between the trachea and vertebral column, was accessible with both modalities.

Lower paratracheal nodes (Station 4) on the right side (4R) were examined between the lower edge of the innominate vein and the lower border of the azygos vein. Localization was achieved by carefully withdrawing from the right main bronchus while visualizing both the azygos vein and the superior vena cava. Differentiation from station 10R was based on the anatomical relationship to the azygos vein, with N1 nodes located below. Left-sided nodes (4L) were evaluated within the area bounded superiorly by the aortic arch and inferiorly by the left pulmonary artery, adjacent to the ligamentum arteriosum. Visualization was achieved bronchoscopically via the left main bronchus and supplemented by EUS, which identified the descending aorta and its transition to the arch.

Aortopulmonary window (Station 5) nodes in this area were examined lateral to the aortic arch and proximal to the origin of the left pulmonary artery. Boundaries were defined by the inferior surface of the aortic arch above and the upper margin of the left pulmonary artery below.

**Figure 2:**
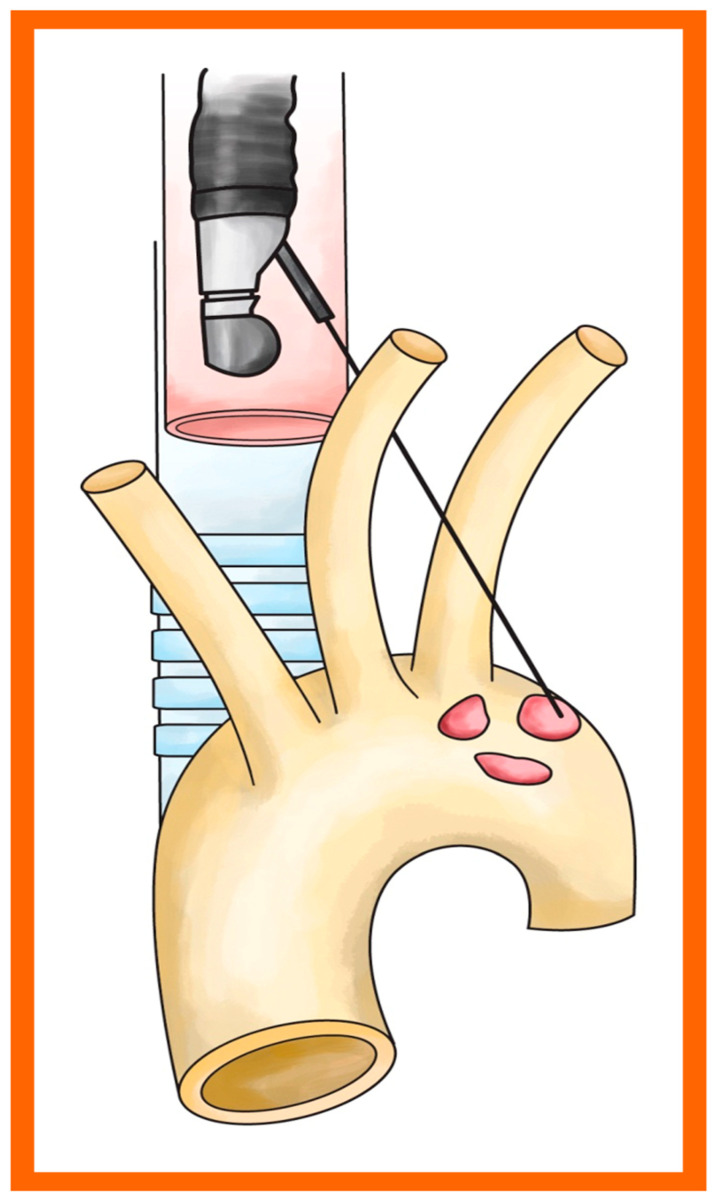
Access to station 6 lymph nodes using endoscopic ultrasound (EUS). Illustration demonstrating transesophageal ultrasound-guided needle access to para-aortic (station 6) lymph nodes. Adapted with permission from Liberman et al. [58]. (copyrights license 6261260987669).

Station 6 lymph nodes are found in front of and to the side of the ascending aorta and aortic arch, below the top edge of the aortic arch, and above its lower edge. These nodes are hard to see with EBUS, but with EUS, we can find them by locating the aortic arch and scanning around it, looking through and above the arch [60]. Newer methods now allow biopsies of lymph nodes in stations 5 and 6, either by crossing the aorta or not, using EUS or EBUS [61,62]. See Figure 2 for reference.

The subcarinal compartment (station 7) is anatomically bounded by the left lower lobe bronchus superiorly on the left and the bronchus intermedius inferiorly on the right. Access is achieved through either bronchial or esophageal routes, with the scope’s direction adjusted according to the chosen approach. In practice, sampling is most commonly performed using the esophageal technique. After inserting the echoendoscope, gradual withdrawal allows visualization of adjacent cardiac and vascular structures, including the left atrium and pulmonary artery. Controlled rotation of the probe aligns the imaging plane with the subcarinal space. Special caution is needed during needle advancement due to its proximity to the pericardium.

Stations 8 and 9 are assessed along the inferior mediastinum in relation to pulmonary venous anatomy and the diaphragm. Station 8 is located in a paraesophageal position between the superior and inferior pulmonary veins, extending upward to the level of the subcarinal bronchi and downward to the diaphragm. Further down, station 9 is situated within the pulmonary ligament, bounded above by the inferior pulmonary vein and below by the diaphragm. Endoscopic orientation depends on sequentially visualizing mediastinal landmarks during controlled withdrawal within the esophagus, with the superior pulmonary vein serving as a key reference point.

Hilar nodal groups are accessed via bronchoscopy. On the right, station 10R is evaluated from the main bronchus just before the upper lobe bronchus, using the azygos vein as the main landmark to define its upper boundary. On the left, station 10L is encountered along the main bronchus beneath the pulmonary artery. Interlobar nodes (station 11) are examined at the level of the secondary carina, which corresponds to the bifurcation of the lobar bronchi on each side.

Evaluation of extrathoracic sites is conducted using the esophageal approach to improve staging completeness. The left adrenal gland is visualized from the gastric position by first identifying the left kidney and spleen; it usually appears as a curved structure located above the kidney. To assess the right adrenal gland, the scope is advanced into the duodenum beyond the pylorus, then the right kidney is located, and the scope is gradually withdrawn to expose the gland superiorly. The celiac axis is evaluated by tracing the abdominal aorta to its first major anterior branch, and the surrounding lymphatic tissue in this area is examined for potential metastatic spread.

A structured sequence has been suggested to help acquire endosonographic skills. For bronchoscopic ultrasound, a common progression includes stations 4L, 7, 10L, 10R, the azygos vein, and 4R. In the esophageal approach, orientation typically begins with the left lobe of the liver and descending aorta, then moves to the left adrenal gland, followed by mediastinal stations such as 7, 4L, and 4R [78].

### 8.2. Needle Technique

To puncture a lymph node, advance the needle quickly. Hold the needle with the thumb pointing down to control its movement. Once inside the node, move the needle in and out, ensuring to see the needle moving within the lymph node rather than moving the node itself. Take two samples without suction to minimize blood contamination. Collect a third sample using negative-pressure suction for cell block, histology, and molecular analysis. This is one approach; there is no strong evidence supporting suction, and a slow-pull method may also be used [78].

### 8.3. Rapid On-Site Evaluation (ROSE)

Regarding the use of ROSE (Rapid Onsite Evaluation), which is a specialized, real-time diagnostic procedure where a cytopathologist or trained professional examines cytologic samples immediately during a needle aspiration biopsy (FNA), it has not been shown to improve diagnostic yield [77,79]. However, if available, ROSE can reduce the number of needle passes and the number of slides prepared during the procedure. ROSE may also help improve technique and reduce the number of procedures [80]. Some studies suggest that ROSE may improve sampling adequacy and diagnostic performance. Physicians using ROSE are able to obtain consistently higher-quality tissue, which helps them diagnose conditions more accurately and increases the likelihood of properly detecting cancer. For instance, ROSE raised the proportion of usable samples by 12%, boosted overall diagnostic accuracy by 14%, and upped the sensitivity for identifying cancer by 10%.

ROSE also typically means fewer needle insertions are needed to collect sufficient tissue, making it less unpleasant for patients. Importantly, it does not extend procedure times or raise complication rates—both remain unchanged.

Results from ROSE align remarkably well with final lab diagnoses, showing a 97% concordance. This reliability lets clinicians make immediate decisions with confidence. These benefits are greatest for people with solid lung tumors or suspected lung cancer, though they are less significant in lymph node evaluations or similar scenarios.

Additionally, ROSE allows for the collection of samples suitable for further tests, such as those needed for personalized lung cancer therapies. Overall, the current research strongly supports ROSE as a valuable option in cases where fast, precise results and high-quality specimens really matter.

A comparative study of cytology slides and cell block preparations demonstrated high diagnostic performance, with similar results observed for both methods. The combination of cytology slides with either core tissue or cell block achieved the highest diagnostic performance, although these approaches required greater resources. The diagnostic yield and accuracy for each preparation method are as follows [81]:

- Cytology slides: 81%, 80%;
- Cell block: 48%, 33%;
- Core tissue: 87%, 99%;
- Cytology slides + core tissue: 80%, 100%;
- Cytology slides + cell block: 86%, 100%.

### 8.4. Needle Gauge Selection

Comparative studies have reported similar specimen adequacy and overall diagnostic yield for 21G and 22G needles in EBUS-TBNA. Some evidence suggests that, when rapid onsite evaluation (ROSE) is available, using a 21G needle may decrease the number of passes needed [82]. Larger-caliber needles have also been linked to improved diagnostic performance in certain situations, including higher detection rates for benign disease (83% vs. 60%) and greater accuracy in histologic subtyping of non-small cell lung cancer (88% vs. 65%) compared to 22G needles [83]. In EUS-guided sampling of solid lesions adjacent to the gastrointestinal tract, current data have not shown a significant difference in diagnostic accuracy between fine-needle aspiration and fine-needle biopsy [84]. Emerging evidence further indicates that 19G EBUS needles may offer technical advantages, including easier maneuverability, an acceptable safety profile, and potentially higher diagnostic yield [85].

Prospective randomized controlled trials have demonstrated no benefit of suction compared to non-suction during needle aspiration, with no observed difference in diagnostic yield or specimen adequacy [86]. Data remain insufficient regarding the impact of suction versus non-suction on the acquisition of molecular markers in NSCLC. For mediastinal staging of potentially operable NSCLC using EBUS-TBNA, optimal results are achieved with three passes per lymph node station, as the diagnostic yield plateaus after three passes [87].

Lymph node characteristics that suggest, but do not definitively indicate, malignancy include round shape, heterogeneous appearance, distinct margins, and the presence of a coagulative necrosis sign [88]. Heterogeneous features are more predictive of malignancy than other characteristics [89]. However, no LN feature can definitively confirm or rule out malignancy. These signs may be particularly useful when multiple LNs are present within the same station, particularly if their size is below the usual cut-off values (e.g., <5 mm) [90]. Color Doppler imaging may also help in choosing the most appropriate target. In particular, vascular patterns such as the bronchial artery inflow sign can help direct needle placement toward nodes that are more suspicious during EBUS-TBNA. Prospective studies suggest that this approach may improve sampling accuracy [91]. reported sensitivity, specificity, positive predictive value, negative predictive value, and diagnostic accuracy of the bronchial artery flow sign using color Doppler as 0.93, 0.64, 0.84, 0.6, and 0.81, respectively. EBUS elastography also helps differentiate benign from malignant lymph nodes. In a prospective study by Sun et al. [92], which included 56 patients with 68 LNs (33 benign and 35 malignant), elastography distinguished benign from malignant LNs with high accuracy. Sensitivity, specificity, positive predictive value, negative predictive value, and accuracy for benign versus malignant LNs were 85.71, 81.82, 83.33, 84.38, and 83.82%, respectively, compared to 91.43, 72.73, 78.05, 88.89, and 82.35%, respectively. Ultrasonographic features of lymph nodes can provide additional clues regarding malignancy. The Canada Lymph Node Score (CLNS) was developed as a simple, weighted, evidence-based score to predict malignancy at the time of EBUS based on real-time ultrasound characteristics.

**Figure 3:**
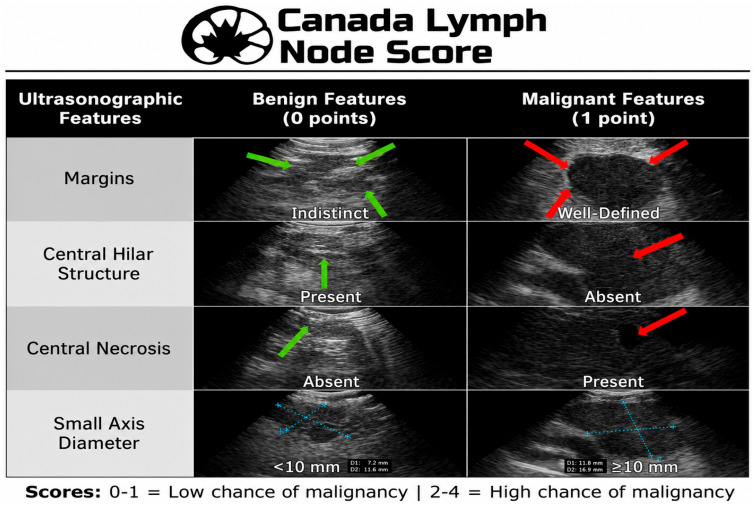
Canada Lymph Node Score for ultrasonographic assessment of mediastinal lymph nodes. The scoring system is based on four sonographic features: nodal margins (indistinct vs. well-defined), central hilar structure (present vs. absent), central necrosis (absent vs. present), and small axis diameter (<10 mm vs. ≥10 mm). Each malignant feature scores 1 point, with total scores ranging from 0 to 4. A score of 0–1 indicates a low probability of malignancy, whereas a score of 2–4 suggests a high probability of malignancy. (copyrights license 6266321270801).

CLN Score Components (1 point each and a maximum score of 4) (Figure 3)

1. Short-axis diameter ≥ 10 mm;
2. Well-defined margins;
3. Absence of central hilar structure;
4. Presence of central necrosis.

Hanna et al. published a prospective multicenter Canadian study including 140 patients with suspected or confirmed lung or esophageal cancer; 300 mediastinal lymph nodes were evaluated with 12 expert raters across 7 centers, and pathology was used as the gold standard. A cut-off score of ≥3 was used to calculate sensitivity, specificity, negative predictive value, and positive predictive value, and showed 31.5%, 96.3%, 65.4%, and 86.5%, respectively.

CLNS is a highly specific tool that helps rule in malignancy and identify which lymph nodes may need repeat biopsy or mediastinoscopy when initial sampling is inconclusive. It can also guide clinicians on prioritizing lymph nodes for biopsy. However, due to lower sensitivity, it should not replace pathological diagnosis [93].

### 8.5. Procedure Competency

Effective training in mediastinal endosonography should include learning anatomy, practicing on simulators, and performing supervised procedures. Outcomes are highly operator-dependent, and learning rates vary. Training exclusively on patients is not ideal, as it can prolong procedures, increase sedation requirements, and raise complication rates [94]. The standard approach is first to learn the six EBUS and EUS landmarks, practice locating them in sequence, and then perform supervised procedures on patients [95,96].

Professional societies, including the American Thoracic Society, the European Respiratory Society, and the American College of Chest Physicians, recommend performing at least 40 procedures to achieve competency and at least 20 cases annually to maintain proficiency [97,98]. Technical skills improve with increased procedural experience, especially after approximately 140 procedures [99,100,101]. These guidelines highlight differences in staging performance among various practitioner groups. Interventional pulmonologists demonstrate greater accuracy compared to general pulmonologists and trainees. In a study by Miller et al., interventional specialists correctly staged 95.5% of cases, while generalists and fellows achieved rates of 44.4% and 30.0%, respectively (*p* < 0.001). Additionally, researchers have examined the agreement between perceived competence and actual performance. Interventional pulmonologists showed the highest level of agreement, whereas less experienced groups exhibited lower concordance levels [102].

## 9. Endosonography in Lymphoma, Sarcoidosis and Infections

The sensitivity of EBUS in diagnosing lymphoma ranges from approximately 57% to 61%. The value of EBUS-TBNA in diagnosing lymphoma remains debated. While EBUS-TBNA can serve as the initial diagnostic modality for lymphomas, its diagnostic yield is low, especially for suspected new cases, particularly Hodgkin’s lymphoma. Negative results from EBUS-TBNA do not rule out lymphoma. Therefore, further interventions such as mediastinoscopy should be considered for patients with a high suspicion of disease following negative or non-diagnostic endosonography [103,104].

A large retrospective study done by Dayan et al. evaluated the effectiveness of endosonography as a first-line diagnostic test for suspected mediastinal lymphoma. Over a ten-year period, 444 patients with suspicious mediastinal lymphadenopathy underwent EBUS, EUS, or combined procedures. Lymphoma was ultimately diagnosed in 77 patients. Approximately 88% of lymphoma cases were diagnosed using endosonographic biopsy alone, avoiding the need for surgery. When adequate samples were obtained, diagnostic sensitivity reached approximately 92%, with very high specificity and predictive value. Additionally, the technique allowed accurate subtype classification in nearly 90% of cases, including both Hodgkin and non-Hodgkin lymphomas. The highest sensitivity was observed in low-grade non-Hodgkin lymphomas. The combined use of EBUS and EUS provided the best diagnostic yield, likely because it allowed access to more lymph node stations. The study also highlighted the importance of ancillary studies like flow cytometry, immunohistochemistry, and molecular testing in improving diagnostic accuracy [105].

Another study investigated the role of transbronchial lung cryobiopsy (cryo-TBB) as a diagnostic tool for pulmonary lymphoproliferative disorders. Cryobiopsy is a bronchoscopic technique that freezes lung tissue before extraction, allowing retrieval of larger and better-preserved specimens. Researchers retrospectively reviewed 970 cryobiopsies performed over seven years and identified 13 cases involving lymphoproliferative disorders.

The results demonstrated a high diagnostic success rate. In 12 out of 13 cases, a precise pathological diagnosis was achieved using cryobiopsy combined with immunohistochemistry and molecular testing. Only one case remained inconclusive, and even a subsequent surgical biopsy failed to clarify it. Importantly, cryobiopsy samples were large enough for advanced analyses such as fluorescence in situ hybridization (FISH) and immunophenotyping. Instant freezing did not damage tissue quality or interfere with molecular studies [37].

Both cryobiopsy and endosonography represent major progress in minimally invasive lymphoma diagnostics. They deliver high diagnostic accuracy, near-zero major complication rates, and strong capability for molecular and immunophenotypic analysis. When performed in experienced centers, these methods should be considered first-line diagnostic strategies before surgical biopsy.

Endosonography is effective for diagnosing granulomas in people with sarcoidosis. In these cases, imaging typically shows clusters of lymph nodes symmetrically arranged around large blood vessels. The structure of the lymph nodes is generally maintained, and a hilum is visible. Endosonography is more sensitive and accurate than simple mucosal biopsies, whether or not a blind transbronchial puncture is used [106,107,108]. A pooled analysis found that EBUS-TBNA had 84% sensitivity and 100% specificity for diagnosing sarcoidosis [97]. Combined EBUS/EUS endosonography is also more accurate (80%) than conventional bronchoscopic biopsies (53%) in these patients [109].

EBUS-TBNA shows 70% sensitivity, 97.2% specificity, and 89.9% accuracy for diagnosing tuberculosis in hilar and mediastinal lymph nodes, based on cytology or histology of caseating granulomas [93]. It also has a higher diagnostic yield than cTBNA for histoplasmosis [74].

A study of 43 HIV-positive patients with mediastinal lymphadenopathy found that combining bronchoalveolar lavage (BAL) with transbronchial biopsy (TBB) gave a 69.8% diagnostic yield. BAL with EBUS-TBNA had an 86% yield, and TBB with EBUS-TBNA reached 88.4%. Tuberculosis was the most common diagnosis, and EBUS-TBNA was more accurate than BAL [104].

Fine-needle aspiration cytology (FNA) is increasingly used to diagnose infections, especially as more patients are immunocompromised. Endosonographic FNA is often the first test when clinical and imaging findings suggest an infectious lesion that can be reached with these methods. However, great care is needed when aspirating mediastinal cysts, since using transbronchial or transesophageal routes can introduce infection. Duplication and foregut cysts have unique echoendoscopic features, including multilayered walls and anechoic contents. If these cysts are uncomplicated, they can usually be diagnosed without a biopsy or its risks.

## 10. Newer Thin Convex EBUS

In a recent ex vivo human lung study, the newer thin convex probe EBUS (with a 5.9 mm tip) demonstrated improved reach and higher success rates compared to the standard convex probe EBUS. It achieved an average maximum reach of 22.1 mm and a further endoscopic visibility range of 10.3 mm. This enhanced miniaturized EBUS probe could selectively assess almost all segmental bronchi, with 98% of the right and 91% of the left segments being accessible. This exceeds the accessibility of the conventional convex probe EBUS, which could only reach 48% of the right and 47% of the left segments. This advancement allows for more precise assessment of N1 nodes and potentially identifies intrapulmonary lesions that are normally inaccessible to the conventional convex probe EBUS without difficulty in needle deployment or sampling [110]. As N1 staging has become more important in the era of preoperative induction therapy in NSCLC, these thinner scopes are becoming vital to clinical decision-making.

## 11. Conclusions

Combined endosonographic techniques (EBUS/EUS) have largely replaced conventional mediastinoscopy as the preferred initial staging modality. Compared with mediastinoscopy, EBUS and EUS are associated with reduced procedural morbidity, shorter recovery time, and lower overall healthcare costs.

The combined EBUS-EUS technique offers superior sensitivity and negative predictive value, allowing for the sampling of multiple stations and distant metastases, including structures below the diaphragm. These techniques are likely to remain central to mediastinal staging. It is important to note that EBUS and EUS should be considered complementary techniques.

The choice of the best minimally invasive approach should be based on the available resources and expertise. In patients with suspicious lymph nodes detected on either CT or PET scans, negative endosonographic findings should be confirmed surgically when clinically indicated.

## Figures and Tables

**Table 1:** Stage groups of the 9th edition of the IASLC Tumor, Node, Metastasis (TNM) classification for lung cancer.

T Category	Descriptor	N0	N1	N2a	N2b	N3
T1a	≤1 cm	IA1	IIA	IIB	IIIA	IIIB
T1b	>1 to ≤2 cm	IA2	IIA	IIB	IIIA	IIIB
T1c	>2 to ≤3 cm	IA3	IIA	IIB	IIIA	IIIB
T2a	Visceral pleura/central invasion	IB	IIB	IIIA	IIIB	IIIB
T2a	>3 to ≤4 cm	IB	IIB	IIIA	IIIB	IIIB
T2b	>4 to ≤5 cm	IIA	IIB	IIIA	IIIB	IIIB
T3	>5 to ≤7 cm	IIB	IIIA	IIIA	IIIB	IIIC
T3	Invasion	IIB	IIIA	IIIA	IIIB	IIIC
T3	Same lobe separate nodules	IIB	IIIA	IIIA	IIIB	IIIC
T4	>7 cm	IIIA	IIIA	IIIB	IIIB	IIIC
T4	Invasion	IIIA	IIIA	IIIB	IIIB	IIIC
T4	Ipsilateral separate tumor nodules	IIIA	IIIA	IIIB	IIIB	IIIC
M1a	Contralateral tumor nodules	IVA	IVA	IVA	IVA	IVA
M1a	Pleural/pericardial effusion or nodules	IVA	IVA	IVA	IVA	IVA
M1b	Single extrathoracic metastasis	IVA	IVA	IVA	IVA	IVA
M1c1	Multiple metastases in one organ	IVB	IVB	IVB	IVB	IVB
M1c2	Multiple metastases in >1 organ	IVB	IVB	IVB	IVB	IVB

**Table 2:** IASLC TNM definitions (9th edition) for primary tumor (T), regional lymph nodes (N), and distant metastasis (M) in lung cancer.

**T: Primary Tumor**	
**Category**	**Definition**
Tx	Primary tumor cannot be assessed
T0	No evidence of primary tumor
Tis	Carcinoma in situ
T1	Tumor surrounded by lung or visceral pleura, or in a lobar or more peripheral bronchus
T1mi	Minimally invasive adenocarcinoma
T1a	Tumor ≤ 1 cm in greatest dimension
T1b	Tumor > 1 cm but ≤2 cm
T1c	Tumor > 2 cm but ≤3 cm
T2	Tumor with any of the following features
T2a	>3 cm but ≤4 cm; or visceral pleura invasion; or involves main bronchus; or associated with atelectasis/obstructive pneumonitis
T2b	>4 cm but ≤5 cm
T3	>5 cm but ≤7 cm; or chest wall invasion; or pericardium/phrenic nerve involvement; or same-lobe tumor nodules
T4	>7 cm; or invasion of mediastinum, heart, great vessels, trachea, esophagus, diaphragm; or tumor nodules in a different ipsilateral lobe
**N: Regional Lymph Nodes**	
**Category**	**Definition**
NX	Regional lymph nodes cannot be assessed
N0	No regional lymph node metastasis
N1	Ipsilateral peribronchial and/or hilar lymph node involvement
N2	Ipsilateral mediastinal and/or subcarinal lymph nodes
N2a	Single-station N2 involvement
N2b	Multi-station N2 involvement
N3	Contralateral mediastinal or hilar, or supraclavicular lymph nodes
**M: Distant Metastasis**	
**Category**	**Definition**
M0	No distant metastasis
M1	Distant metastasis present
M1a	Pleural/pericardial disease or contralateral lung nodules
M1b	Single extrathoracic metastasis
M1c	Multiple extrathoracic metastases
M1c1	Multiple metastases in a single organ
M1c2	Multiple metastases in multiple organs

## Data Availability

Data sharing is not applicable to this article as no new data were created or analyzed in this study.
